# Lysozyme Counteracts β-Lactam Antibiotics by Promoting the Emergence of L-Form Bacteria

**DOI:** 10.1016/j.cell.2018.01.021

**Published:** 2018-02-22

**Authors:** Yoshikazu Kawai, Katarzyna Mickiewicz, Jeff Errington

**Affiliations:** 1 Centre for Bacterial Cell Biology, Institute for Cell and Molecular Biosciences, Newcastle University, Newcastle upon Tyne NE2 4AX, UK

**Keywords:** bacterial genetics, bacterial cell biology, macrophage, *Bacillus subtilis*, *Staphylococcus aureus*, antibiotic action, penicillin, lysozyme, bacterial cell wall, antibiotic resistance

## Abstract

β-lactam antibiotics inhibit bacterial cell wall assembly and, under classical microbiological culture conditions that are generally hypotonic, induce explosive cell death. Here, we show that under more physiological, osmoprotective conditions, for various Gram-positive bacteria, lysis is delayed or abolished, apparently because inhibition of class A penicillin-binding protein leads to a block in autolytic activity. Although these cells still then die by other mechanisms, exogenous lytic enzymes, such as lysozyme, can rescue viability by enabling the escape of cell wall-deficient “L-form” bacteria. This protective L-form conversion was also observed in macrophages and in an animal model, presumably due to the production of host lytic activities, including lysozyme. Our results demonstrate the potential for L-form switching in the host environment and highlight the unexpected effects of innate immune effectors, such as lysozyme, on antibiotic activity. Unlike previously described dormant persisters, L-forms can continue to proliferate in the presence of antibiotic.

## Highlights

• On isotonic media, β-lactams can kill by non-lytic mechanisms
• Lysozyme protects from β-lactam killing by enabling L-form escape
• Immune cells can convert bacteria to L-forms, protecting them from β-lactams
• L-forms could act as persister cells that grow in the presence of β-lactams

## Graphical Abstract

**Figure:**
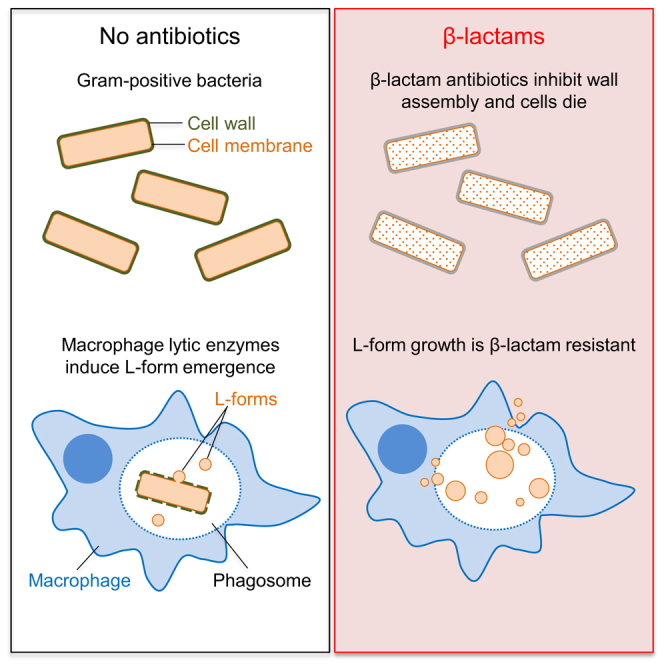

## Teaser

Innate immune effectors with lytic activity can paradoxically promote a switch to an antibiotic resistant form that allows bacteria to proliferate in host environments.

## Introduction

**Figure 1:**
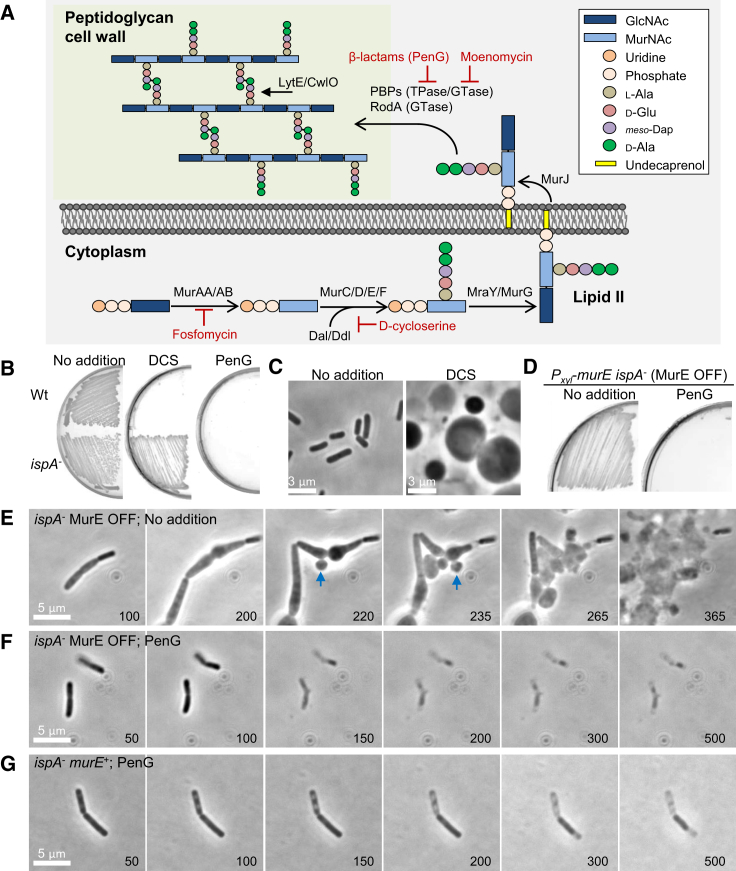
PenG Prevents the L-Form Switch from the Walled State (A) Schematic representation of peptidoglycan (PG) synthesis in *B*. *subtilis* and its inhibition by antibiotics. The PG wall is built from long glycan strands composed of N-acetylmuramic acid (MurNAc) and N-acetylglucosamine (GlcNAc) crosslinked to each other by short peptide bridges. The precursor for PG is initially synthesized in the cytoplasm by the action of MurAA, MurAB, MurC, MurD, MurE, and MurF enzymes. MurNAc-pentapeptide is coupled to a membrane carrier, undecaprenyl pyrophosphate, by MraY, and GlcNAc is added by MurG to form lipid II, which is then transferred to the outside of the cytoplasmic membrane. Newly synthesized PG is incorporated into the existing PG meshwork by a combination of transglycosylation and transpeptidation reactions catalyzed by penicillin-binding proteins (PBPs) and RodA. The antibiotics fosfomycin and D-cycloserine inhibits MurA and Ddl, respectively. The β-lactam antibiotics including penicillins (e.g., penicillin G) and cephalosporins (e.g., cephalexin) target the PBPs. (B–D) Effects of antibiotics on L-form switch. (B) *B*. *subtilis* strains wild-type (168CA) and *ispA* mutant (RM81) were grown on NA/MSM plates with or without 200 μg/mL D-cycloserine (DCS) (with 1 μg/mL of FtsZ inhibitor 8j to prevent the rare reversion to walled cells) or 200 μg/mL penicillin G (PenG) at 30°C for 2–3 days. (C) PC micrographs of *ispA* mutant cells with or without DCS taken from the cultures shown in (B). (D) *B*. *subtilis* L-form strain (LR2; *P*_*xyl*_-*murE ispA*^−^) was grown on NA/MSM plates with or without PenG in the absence of xylose. (E–G) Effects of PenG on L-form emergence from the parental walled cells in LR2 (*P*_*xyl*_-*murE ispA*^−^; E and F) and RM81 (*ispA*^−^*murE*^+^; G). Individual frames are extracted from example movies of time-lapse experiments (see also Movies S1 and S2). Numbers in the bottom right corner of each frame represent time (min) elapsed in the movies. Examples of L-form emergence are shown by blue arrows. See also Figures S1 and S2.

The cell wall is one of the defining structures of bacterial cells, and the main component is a normally essential polymer called peptidoglycan (PG) (Höltje, 1998, Typas et al., 2011). The PG precursor molecule, called lipid II, is made in the cytosol via a well conserved biochemical pathway, then transferred to the outside of the cytoplasmic membrane, where it is assembled by glycosyltransferase (GTase) and transpeptidase (TPase) enzymes (Figure 1A). The key TPases are called penicillin-binding proteins (PBPs) because they are targeted by penicillin and other β-lactam antibiotics (Lovering et al., 2012). β-lactams are one of the oldest and still most widely used antibiotics. They prevent cell wall assembly and it is generally considered that this kills bacteria by explosive cell lysis. However, classical microbiological culture conditions are mainly hypotonic and under osmoprotective (isotonic) conditions lysis is largely suppressed, although the cells are prevented from growing and die, possibly through other mechanisms such as oxidative damage (Dwyer et al., 2015, Kawai et al., 2015) or futile cycling of the cell wall precursor pathway (Cho et al., 2014).

In light of the importance of the wall, it is surprising that many bacteria are able to switch into a wall-deficient state called the L-form (Klieneberger, 1935), which is completely resistant to most antibiotics working specifically on cell wall synthesis, particularly β-lactams. Most classically described L-forms were observed in samples obtained from humans, animals, and plants and also identified as antibiotic resistant organisms isolated in association with a wide range of infectious diseases (Allan et al., 2009, Domingue and Woody, 1997, Errington et al., 2016). However, their possible role in chronic or recurrent infections remains controversial. Crucially, the molecular events underlying switching to and from the L-form state in the host environment are presently unclear.

The Gram-positive bacterium *Bacillus subtilis* has been reported to switch into the L-form state under laboratory conditions (Leaver et al., 2009), as well as in plants (Ferguson et al., 2000). We have recently developed a tractable system for studying the cell biology and genetics of *B. subtilis* L-forms and found key genetic changes associated with the initial switch from the walled to the L-form state (Domínguez-Cuevas et al., 2012, Kawai et al., 2015, Leaver et al., 2009, Mercier et al., 2013). Those studies highlight that L-forms do not require PG synthesis or the FtsZ-based division machine, both of which are normally essential, for their proliferation (Adams and Errington, 2009). Instead, L-form proliferation is brought about by an increased rate of membrane synthesis, leading to an increased membrane surface area to volume ratio, which drives cell shape deformations that lead to spontaneous scission (Mercier et al., 2013). The excess membrane synthesis can be generated by directly activating the fatty acid membrane synthetic pathway, or indirectly, by shutting down lipid II precursor pathway, which works via an as yet uncharacterized mechanism (Mercier et al., 2013). Antibiotics that block lipid II precursor synthesis, such as fosfomycin and D-cycloserine, also rapidly and efficiently induce the L-form switch in a wide range of bacteria, including the pathogenic Firmicute *Staphylococcus aureus*, under laboratory conditions (Mercier et al., 2014).

Historically, β-lactam antibiotic treatments have been used to promote the L-form state in osmotically supportive medium (e.g., in the presence of sucrose) in many laboratories; however, this is usually a tedious process with a low success rate and requires long term and repeated passage (Allan, 1991). In our early experiments with *B. subtilis* L-forms, we found that penicillin treatment surprisingly prevents the switch from walled to L-form states (Leaver et al., 2009). We subsequently started to characterize the L-form switch in further detail and found that escape of the protoplast from the enveloping cell wall was an important intermediate step that could be influenced by various antibiotics and lysozyme (Domínguez-Cuevas et al., 2012). However, at that time we were unaware of the confounding role of oxidative stress in L-form growth (Kawai et al., 2015). Because we were not tracking the presence/absence of SNPs that might affect oxidative stress, interpretation of the effects of other factors on L-form growth was problematical. We now show that in *B. subtilis* and a wide range of Gram-positive bacteria (but not the Gram-negative *Escherichia coli*), β-lactams fail to induce efficient L-form switching and that this is likely because inactivation of class A, bifunctional PBPs results in inhibition of the autolytic enzymes responsible for escape of the cell protoplast from the parental walled cell. Crucially, addition of an exogenous PG hydrolase, such as lysozyme, enables L-form escape and thereby protects from cell killing by the antibiotics. Using an animal model, *Galleria mellonella*, we show that the L-form transition can be initiated in a host environment, presumably by host lytic enzymes. Finally, we reproduce these effects in an *in vitro* mammalian macrophage system and show that under these conditions macrophages can actually protect input walled cells from penicillin killing. The results have important implications for our understanding of β-lactam antibiotic activity under physiologically relevant conditions, especially for how bacteria can evade antibiotic action utilizing innate immune effectors of host cells. They also stress that the killing effects of antibiotics can vary dramatically depending on the culture conditions, including the level of osmoprotection and presence of lytic enzymes.

## Results

### PenG Inhibits the Emergence of L-Forms from Parental Walled Cells

We have previously shown that inhibition of the lipid II pathway promotes L-form formation and proliferation on isotonic (i.e., sufficiently osmoprotective to support L-form growth) media in a wide range of bacteria (Mercier et al., 2013, Mercier et al., 2014). *Bacillus subtilis* is presently the best characterized model for L-form biology (Errington et al., 2016). As shown in Figures 1B and 1C, *B. subtilis* cells grew with a typical rod-shaped morphology on isotonic plates with no antibiotic (No addition). However, when the rods were streaked on plates with D-cycloserine (DCS), which inhibits D-alanine-D-alanine ligase in the lipid II pathway (Figure 1A), they switched to spherical wall-deficient L-forms (Figures 1B and 1C, DCS). Note that under aerobic conditions, *B. subtilis* L-form growth also requires a mutation in a gene such as *ispA* to avoid cell lysis due to increased oxidative damage in wall-deficient cells (Kawai et al., 2015), so all of the experiments with *B. subtilis* herein involved strains bearing an *ispA* mutation. In contrast, growth was severely impaired when the rods were streaked on isotonic plates with β-lactam antibiotics penicillin G (PenG) or cephalexin (Figures 1B, PenG, and S1A, cephalexin), which inhibit PG assembly (Figure 1A). A few colonies eventually emerged but only after 3 days or more of incubation.

**Figure S1:**
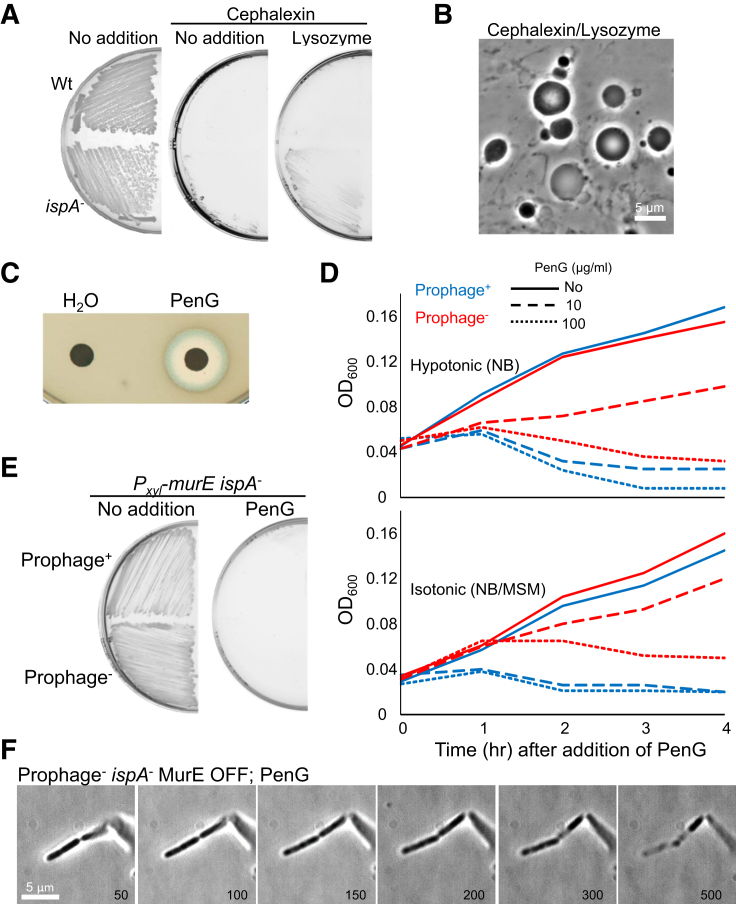
Effects of Prophages and Lysozyme on β-Lactam Killing in *B. subtilis*, Related to Figures 1 and 2 (A and B) Effects of Lysozyme on *B. subtilis* L-form growth in the presence of cephalexin. (A), *B*. *subtilis* strains wild-type (168CA) and *ispA* mutant (RM81) were grown on NA/MSM plates in the presence of 100 μg/ml cephalexin, with or without 100 μg/ml lysozyme, as indicated, at 30°C for 2-3 days. (B), PC micrographs of *ispA* mutant cells taken from the plates in the presence of cephalexin and lysozyme shown in (A). (C) Disc diffusion assay of a *B. subtilis* strain [CU267 (Ø105MU3)] carrying *lacZ* reporter for prophage encoded gene on MSM plates containing X-gal using 0.02 μg PenG per disc. (D) Growth profiles of *B. subtilis ispA* mutant in the presence (strain RM81; blue line) or absence of prophages (strain YK2313; red line) under hypotonic (NB) and isotonic NB/MSM medium with (red line) or without PenG. *B. subtilis* strains were grown at 30°C without shaking. (E) Effects of prophages on *B. subtilis* L-form growth in the presence of PenG. *B*. *subtilis* L-form strains (LR2; *P*_*xyl*_-*murE ispA*^-^, and YK2314; *P*_*xyl*_-*murE ispA*^-^ prophage^-^) was grown on NA/MSM plates with or without 200 μg/ml PenG in the absence of xylose.

As reported previously, *B. subtilis* strain LR2, which carries an *ispA* mutation and a repressible *P_xyl_-murE* construct (that allows control over lipid II synthesis), also undergoes an efficient L-form switch when lipid II synthesis is repressed under isotonic conditions (Leaver et al., 2009, Mercier et al., 2013) (Figure 1D, no addition). Once initiated, this L-form mode of proliferation confers complete resistance to most antibiotics acting on PG biogenesis, including PenG (Leaver et al., 2009, Mercier et al., 2013). However, the initiation of L-form growth of the LR2 strain was strongly inhibited in the presence of PenG (Figure 1D, PenG), suggesting that PenG prevents an earlier step in the transition from parental walled rods to L-forms.

We developed time-lapse microscopy methods to visualize the L-form switch from parental rods in the LR2 strain. Figure 1E and Movie S1 show a typical time course. When lipid II synthesis was shut down (MurE OFF), walled rods continued to increase in size and then began to bulge and deform. L-forms then emerged (blue arrows at 220 and 235 min), which propagated in the classical manner (involving a range of unusual tubulation and blebbing events generating a heterogeneous mix of cell sizes and shapes). However, in the presence of PenG (Figure 1F; Movie S2), even though precursor synthesis was shut down, the characteristic bulging phenotype and L-form emergence were largely prevented. Instead, the rods stopped growing and then gradually became phase-pale (over ∼150 min), indicative of cell death.

**Figure S2:**
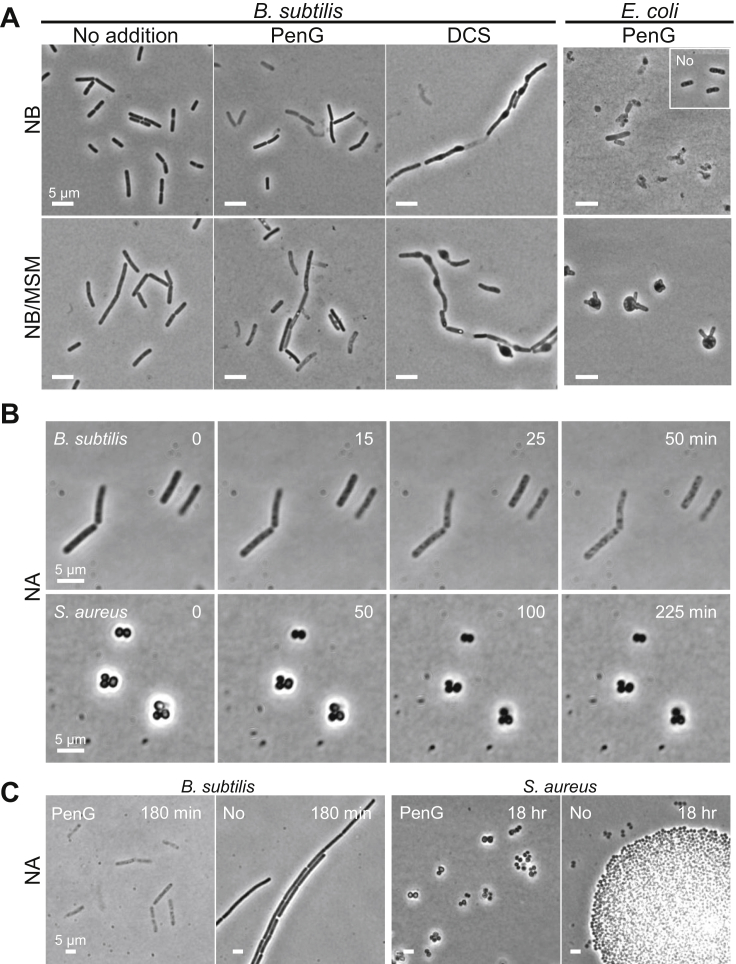
Effects of Isotonicity on β-Lactam Killing in *B. subtilis*, *S. aureus*, and *E*. *coli*, Related to Figures 1 and 3 (A) *B. subtilis* strain RM81 (*ispA*^-^) or *E. coli* wild-type strain BW25113 was grown in hypotonic (NB) or isotonic (NB/MSM) medium with or without 100 μg/ml PenG, or D-cycloserine (DCS) at 30°C for 3-4 hours. (B and C) Effects of PenG on growth of *B. subtilis* (RM81, *ispA*^-^) or *S. aureus* (RN4220, wild-type) under hypotonic conditions. (B), Exponentially growing bacterial cells were placed on NA with 200 μg/ml PenG for time lapse microscopy. Numbers in the top right corner of each frame represent time (min) from time lapse microscopy. (C), Examples of bacterial cells treated with or without PenG.

In passing, it is useful to note that this β-lactam-induced growth arrest and absence of overt death by explosive lysis (Figure 1F) is not just seen under osmoprotective conditions, as here. Figure S2 shows that even under hypotonic conditions, *B. subtilis* (and another Gram-positive organism, *Staphylococcus aureus*) did not change shape significantly for long periods after PenG treatment. This contrasts with the effects of these antibiotics on the Gram-negative bacterium *Escherichia coli*, which underwent bulging and dramatic shape changes in both hypo- and isotonic media (Figure S2; see also below). These differences between Gram-positive and Gram-negative bacteria were first noted decades ago (Rodríguez-Tébar et al., 1982), but this does not seem to have been widely appreciated.

We also note that some of the loss of cell content upon PenG treatment was likely due to the induction of prophage(s) by PenG (Figure S1C). Indeed, the rate at which PenG-treated cells became phase-pale (Figure S1F) or lysed in liquid culture (Figure S1D) was substantially reduced in a strain from which prophages have been removed. Prophage induction by β-lactam action has been described previously for *S. aureus* (Maiques et al., 2006). Because prophages are relatively common in nature, this may be a widespread mechanism of β-lactam killing that has previously been underappreciated. However, in spite of this caveat, we found that PenG still blocked L-form emergence by the MurE OFF condition in the prophage-less background (Figure S1E); the cells evidently died but without significantly changing shape (Figure S1F). We do not fully understand the mechanism of PenG-induced cell death in these experiments, but it may be due to oxidative stress (Foti et al., 2012, Kawai et al., 2015, Kohanski et al., 2007). It is probably not due to futile cycling, as occurs in *E. coli* (Cho et al., 2014) because it still occurred in experiments in which the cells were also inhibited for PG precursor synthesis (Figure 1D). A similar growth arrest and cell death was also observed in a *murE^+^* background with PenG (Figure 1G). It appears that inhibition of the PG precursor pathway not only promotes the L-form mode of proliferation but also induces the emergence of L-forms from parental walled rods, whereas PenG severely impairs L-form emergence.

### Lytic Activity Is Required for L-Form Emergence

**Figure 2:**
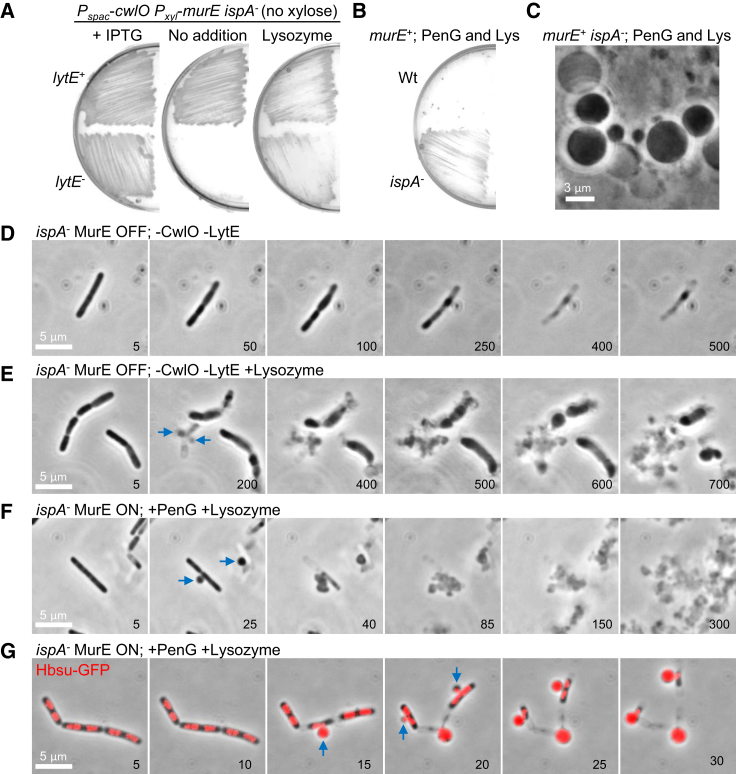
PenG Inhibits L-Form Emergence by Preventing Autolytic Activity (A) Effects of autolytic activity on L-form growth. *B*. *subtilis* strains YK2288 (*lytE*^+^; *P*_*xyl*_-*murE ispA*^−^*P*_*spac*_-*cwlO*) and YK2289 (*lytE*^−^; *P*_*xyl*_-*murE ispA*^−^ Δ*lytE P*_*spac*_-*cwlO*) were grown on NA/MSM plates (no xylose) with or without 0.5 mM IPTG, and 100 μg/mL chicken egg white lysozyme at 30°C for 2–3 days. (B and C) Effects of lysozyme on L-form emergence in the presence of PenG. (B) *B*. *subtilis* strains wild-type (168CA) and *ispA* mutant (RM81) were grown on NA/MSM plates containing 200 μg/mL PenG and lysozyme (Lys) at 30°C for 2–3 days. (C) PC micrographs of *ispA* mutant cells taken from the cultures shown in (B). (D and E) Effects of autolytic enzymes (LytE and CwlO) on L-form emergence from the parental walled cells in *B*. *subtilis* strain (YK2289; *P*_*xyl*_-*murE ispA*^−^ Δ*lytE P*_*spac*_-*cwlO*) in the presence (E) or absence (D) of 20 μg/mL chicken egg white lysozyme. (F) Exogenous lysozyme promotes L-form emergence in the presence of PenG in a *murE*^*+*^ background (strain RM81; *ispA*^−^). (G) Nucleoid dynamics during the L-form emergence from the walled cells in *B*. *subtilis* strain YK2294 (*ispA*^−^*amyE*::*hbsU*-*gfp*) in the presence of PenG and lysozyme. Numbers in the bottom right corner of the time-lapse frames in (D)–(G) represent time (min) elapsed. Examples of L-form emergence in (E)–(G) are shown by blue arrows. See also Figures S1 and S3 and Movies S3 and S4.

We previously reported that efficient L-form emergence in *B. subtilis* requires mutation of a cell wall homeostasis regulator, *walR*, which controls the synthesis and activity of various autolytic enzymes (Domínguez-Cuevas et al., 2012). Consistent with the idea that this mutation works through increased autolytic enzyme activity, elimination of the LytE and CwlO endopeptidases that are essential for normal growth of *B. subtilis* (Hashimoto et al., 2012) strongly inhibited L-form growth of the LR2 strain on isotonic plates (Figure 2A, no addition, *lytE*^−^). Time-lapse microscopy revealed that in the combined absence of LytE and CwlO, the cells did not bulge and L-forms failed to emerge (Figure 2D). Instead, the rods stopped growing and then died without significant shape changes, similar to the effects of PenG on the L-form switch described above (Figure 1F).

As anticipated from our previous work (Domínguez-Cuevas et al., 2012), the addition of exogenous lysozyme rescued the L-form growth defect caused by repression of *lytE* and *cwlO* (Figure 2A, lysozyme). Time-lapse microscopy showed that lysozyme induced the emergence of small L-forms from rods (Figure 2E, blue arrows at 200 min; Movie S3) and they propagated similarly to L-forms in the parental (*lytE*^*+*^, *cwlO*^*+*^) strain (Figure 1E), supporting the idea of a key role for ongoing autolytic activity in L-form emergence induced by inhibition of the lipid II pathway.

If the failure in L-form emergence caused by PenG was due to an antibiotic-induced block in the action of cell wall hydrolases, exogenous lysozyme should also overcome the inhibitory effect of PenG on L-form emergence. To test this, we streaked an *ispA* mutant (wild-type for PG precursor synthesis) on isotonic plates containing both PenG and lysozyme and found that L-form growth occurred efficiently even without *murE* repression (Figures 2B and 2C). Similar results were obtained by time-lapse microscopy (Figure 2F; Movie S4): blue arrows at 25 min point to the emergence of L-forms from rods, which went on to make micro colonies of typical L-form cells. Addition of lysozyme also promoted L-form growth in the presence of a different β-lactam antibiotic, cephalexin (Figures S1A and S1B). Note that although cephalexin has some specificity for a division-associated transpeptidase in many organisms, it nevertheless binds well to the major class A PBP1 of *B. subtilis* at sub 1 μg/mL concentration (Sassine et al., 2017).

**Figure S3:**
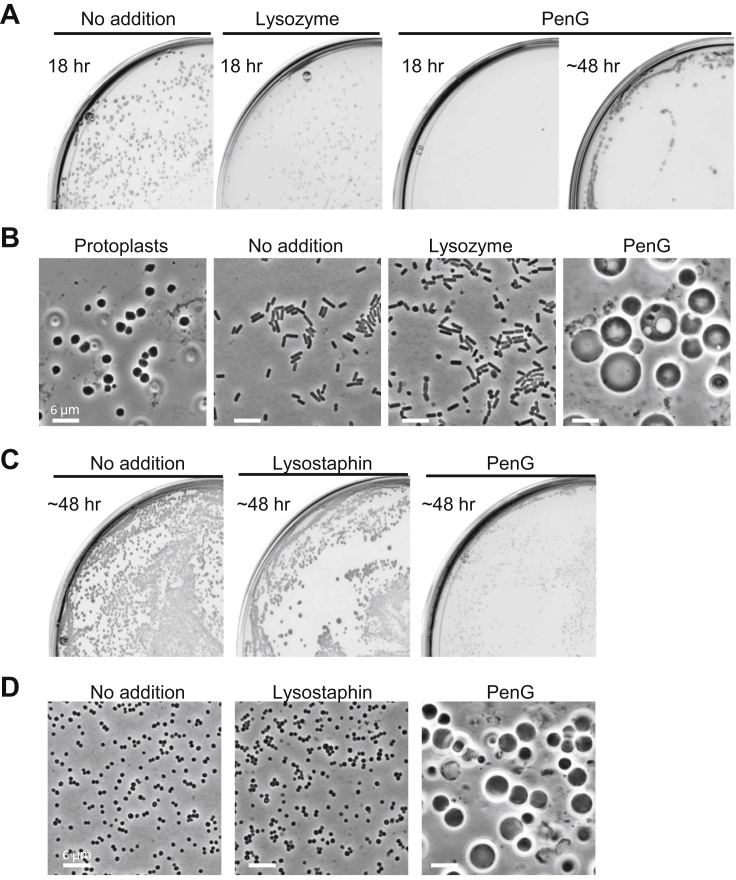
PenG Is Required for L-Form Growth on Osmoprotective Plates, Related to Figures 2 and 4 (A and B) (A), Protoplasts of *B. subtilis* strain RM81 (*ispA*^-^) prepared in NB/MSM with 100 μg/ml lysozyme were plated on NA/MSM with or without 200 μg/ml lysozyme, or 200 μg/ml PenG, as indicated, and incubated at 30°C. (B), PC micrographs of protoplasts prepared in NB/MSM by lysozyme treatment (left), and cells with or without lysozyme, or PenG were taken from the plates shown in panels (A). (C and D), (C), Protoplasts of *S. aureus* wild-type strain (RN4220) prepared in NB/MSM with 5 μg/ml lysostaphin were plated on NA/MSM with or without 5 μg/ml lysostaphin, or 200 μg/ml PenG, as indicated, and incubated at 30°C. (D), PC micrographs of cells with or without lysozyme, or PenG were taken from the plates shown in panels (C).

The above experiment raised the possibility that lysozyme alone might be sufficient to establish and maintain L-form growth. To test this, we made protoplasts (to avoid the need for the protoplast to escape from the cell wall sacculus) of *B. subtilis* and *S. aureus*, which were then plated in the presence of lysozyme (or lysostaphin, an endopeptidase that is a more effective hydrolytic enzyme for *S. aureus* PG) (Wu et al., 2003), or PenG (Figure S3; see also below). As expected, robust L-form growth occurred in the presence of PenG. However, in the presence of lytic enzyme, most of the cell growth visible appeared to be in the walled form (Figure S3B, “Lysozyme”), albeit with a degree of shape distortion and uneven growth. We suggest that because these lytic enzymes do not block *de novo* PG synthesis the cells go through continual wall regeneration and turnover until the lysozyme activity is overwhelmed or used up. This shows that, in practice, cell wall synthesis needs to be blocked (either precursor synthesis or assembly) to enable prolonged L-form growth.

Interestingly, in cells bearing a GFP-fusion to a global DNA binding protein HbsU, it was possible to visualize nucleoids passing out of the parent walled cells during L-form emergence (Figure 2G, blue arrows at 15 and 20 min). Perhaps efficient L-form emergence requires cell wall lesions large enough to accommodate exit of the intact nucleoid.

### Class A PBPs Are Required for Autolytic Activity during the L-Form Switch

**Figure 3:**
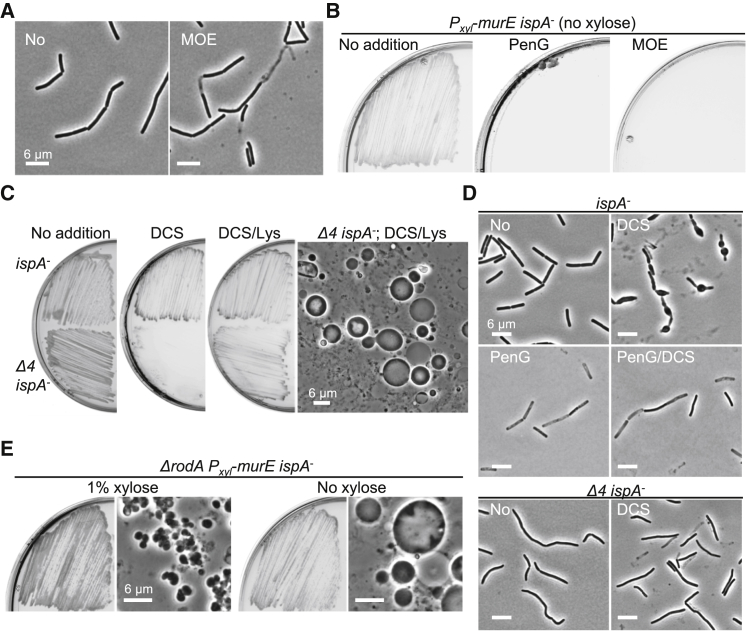
Absence of Class A PBPs Inhibits the L-Form Switch (A and B) Effects of moenomycin on the L-form switch in *B. subtilis*. (A) *B. subtilis* strain RM81 (*ispA*^−^) were grown in NB/MSM with or without 50 μg/mL moenomycin (MOE) at 30°C for 2–3 hr without shaking. (B) *B*. *subtilis* L-form strain (LR2; *P*_*xyl*_-*murE ispA*^−^) was grown on NA/MSM plates with or without 200 μg/mL PenG or 50 μg/mL MOE in the absence of xylose at 30°C for 2–3 days. (C and D) Effects of class A PBPs on L-form switch in *B. subtilis*. (C) *B. subtilis* strain RM81 (*ispA*^−^) and YK2344 (*Δ4 ispA*^*−*^) were grown on NB/MSM with or without 200 μg/mL D-cycloserine (DCS) in the presence or absence of 100 μg/mL lysozyme (Lys) at 30°C for 2–3 days. PC micrograph image of *B. subtilis* L-forms of YK2344 (right) was taken from the plate with D-cycloserine and lysozyme (DCS/Lys). (D) *B. subtilis* strain RM81 (*ispA*^−^) and YK2344 (*Δ4 ispA*^*−*^) were grown in NB/MSM with or without 100 μg/mL PenG and/or D-cycloserine (DCS) at 30°C for 3–4 hr without shaking. (E) Effects of RodA on L-form switch in *B. subtilis*. *B*. *subtilis* strain YK1354 (*ΔrodA P*_*xyl*_-*murE ispA*^−^) was grown on NA/MSM plates in the presence or absence of 1% xylose at 30°C for 2–3 days. PC micrographs of *B. subtilis* cells were taken from the plates. See also Figure S2.

The above results showed that PenG, which inactivates the TPase activity of multiple PBPs, triggers an immediate arrest in cell growth (Figures 1F and 1G). The class A PBPs, of which *B. subtilis* has 4, also possess PG glycosyltransferase (GTase) activity, which is inhibitable by the antibiotic moenomycin (MOE) (van Heijenoort et al., 1987) (Figure 1A). Figure 3A shows that MOE had a similar effect to PenG, in rapidly arresting growth with no significant shape change, and abolished L-form generation (Figure 3B). Similarly, a mutant deleted for all four genes encoding class A PBPs (*ponA*, *pbpD*, *pbpF*, and *pbpG*; “*Δ4*” mutant) was severely inhibited for L-form switching by DCS (Figure 3C, DCS). Again, this inhibition was efficiently reversed by the addition of lysozyme (Figure 3C, DCS/Lys). Microscopic examination of cells treated with DCS revealed that the *Δ4* mutant did not expand or bulge (Figure 3D, *Δ4 ispA*^−^, DCS), similar to the control strain treated with PenG (Figure 3D, *ispA*^−^, PenG/DCS). Thus, class A PBPs are required, directly or indirectly, for the cell wall hydrolytic activity that enables bulging and the L-form switch.

Recently, it was discovered that RodA, a central component of the MreB-dependent cell elongation complex, also has GTase activity, and this may be the major GTase required for cell morphogenesis (Cho et al., 2016, Emami et al., 2017, Meeske et al., 2016). However, deletion of RodA had no discernable effect on the L-form transition (Figure 3E), suggesting that the arrest of autolytic activity by β-lactams is a specific effect of inhibition of the class A PBP PG synthesis pathway.

### Lysozyme Counteracts PenG under Osmoprotective Conditions in Various Gram-Positive Bacteria

**Figure 4:**
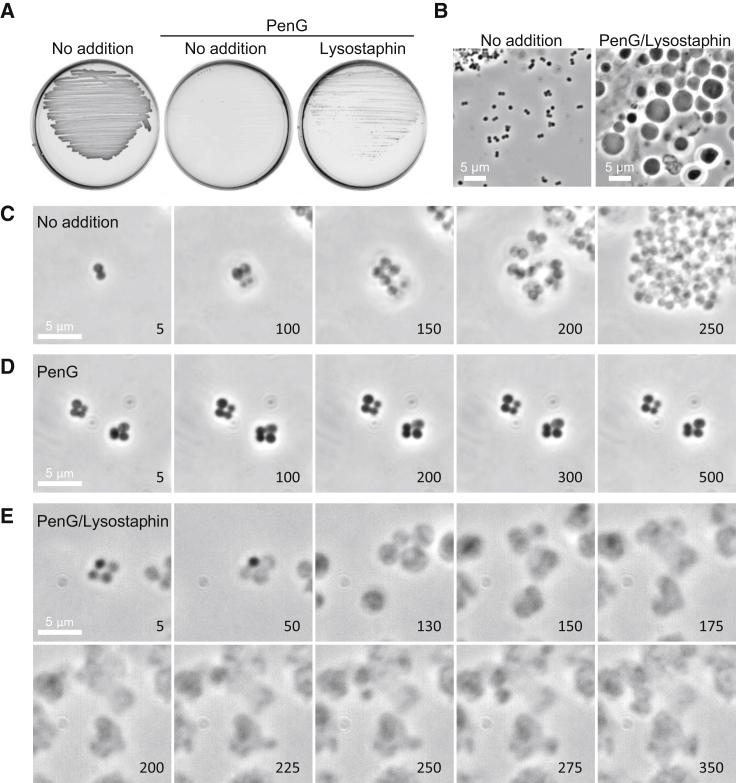
Exogenous PG Hydrolase Promotes *S. aureus* L-Form Switch (A) Effects of PenG and lysostaphin on L-form emergence in *S. aureus*. *S. aureus* strain (RN4220) were grown on NA/MSM plates with or without 200 μg/mL PenG and 2 μg/mL lysostaphin at 30°C for 2–3 days. (B) PC micrographs of *S. aureus* cells in the presence or absence of PenG and lysostaphin taken from the cultures shown in (A). (C–E) Effects of lysostaphin on L-form emergence in the presence of PenG. Exponentially growing *S*. *aureus* walled cells were placed on NA/MSM with PenG (D), both PenG and lysostaphin (E), or without supplements (C) for time-lapse microscopy. Individual frames are extracted from Movies S5 and S6. Numbers in the bottom right corner of each frame represent time (min) elapsed in the movies. Note that the walled *S. aureus* cells pile on top of each other in microcolonies so that the individual cells can become blurred (C). However, in contrast to L-forms (E) the walled cells retain their small uniform shape. See also Figures S3, S4, and S5 and Movie S7.

**Figure S4:**
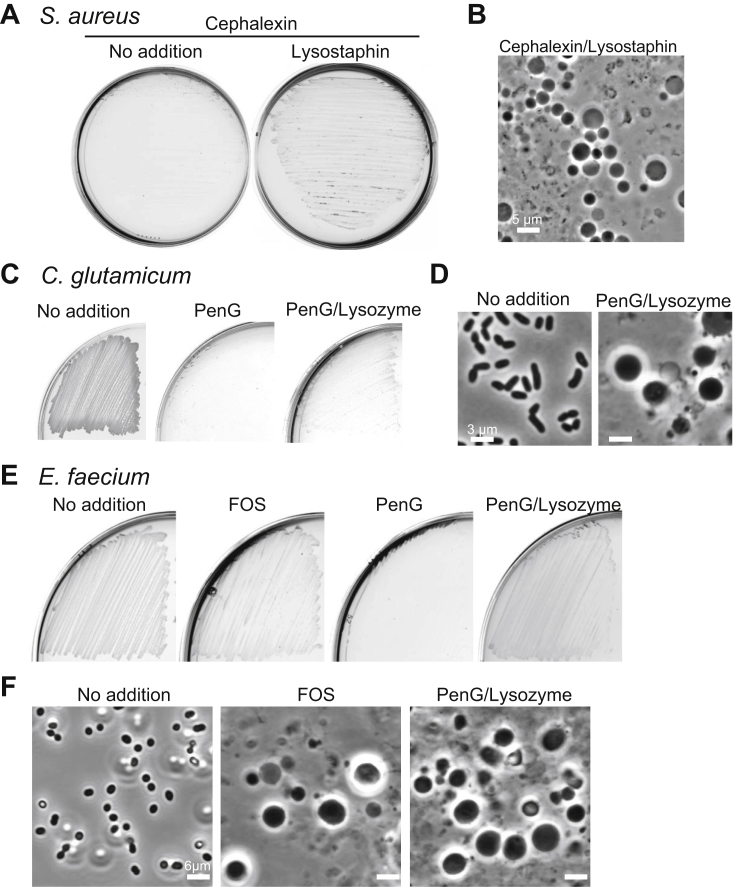
L-Form Switch by Exogenous PG Hydrolase in the Gram-Positive Bacteria, Related to Figure 4 (A and B) Effects of lysostaphin on *S. auleus* L-form growth in the presence of cephalexin. (A), *S*. *aureus* strain (RN4220) was streaked on NA/MSM plates with or without 100 μg/ml cephalexin, and 2 μg/ml lysostaphin, as indicated, and incubated at 30°C for 2-3 days. (B), PC micrographs of cells with lysostaphin and cephalexin taken from the plates shown in (A). (C and D) Effects of lysozyme on *C. glutamicum* L-form growth in the presence of PenG. (C), *C. glutamicum* strain ATCC13032 was streaked on NA/MSM plates in the presence (middle and right) or absence of 200 μg/ml PenG, and 100 μg/ml lysozyme, as indicated, and incubated at 30°C for 2-3 days. (D), PC micrographs of cells taken from the plate shown in (C). (E and F), *E. faecium* L-form growth induced by fosfomycin, or PenG and Lysozyme. (E), *E. faecium* strain ATCC19434 was streaked on Müeller-Hinton/MSM plates with 200 μg/ml fosfomycin (FOS), 200 μg/ml PenG or PenG and 100 μg/ml lysozyme, or without antibiotics (No addition), and incubated at 30°C for 2-3 days. (F), PC micrographs of cells with fosfomycin (middle), PenG and lysozyme (right) or without antibiotics (left) taken from the plates shown in (E).

We previously reported that blocking the PG precursor pathway by fosfomycin or D-cycloserine, which inhibit the MurA or Ddl proteins, respectively (Figure 1A), promotes efficient L-form growth in at least two other Gram-positive organisms, the pathogenic *Firmicute S. aureus* and the *Actinobacterium Corynebacterium glutamicum* (Mercier et al., 2014). However, again, the L-form switch did not occur with PenG (Figures 4 and S4). Figures S4E and S4F show that these findings hold also for another pathogenic *Firmicute*, *Enterococcus faecium*. Furthermore, for all of these organisms, exposure to an exogenous PG hydrolytic enzyme again promoted L-form growth in the presence of PenG (Figures 4 and S4), as shown above for *B*. *subtilis*. Although growth of *S. aureus* L-forms was slightly slower than for *B. subtilis*, L-form colonies emerged within 2 days of incubation on isotonic plates with PenG (or cephalexin) and lysostaphin (Figures 4A, 4B, S4A, and S4B). Time-lapse microscopy showed that PenG blocked the proliferation of *S. aureus* walled cells (Figures 4C and 4D; Movie S5). However, in the presence of lysostaphin, the cells became pale in phase contrast images, presumably due to cell wall degradation (Figure 4E; Movie S6). The cells then started to grow and change shape, initiating a typical mode of L-form proliferation. Note that time-lapse imaging with *S. aureus* is challenging because the L-forms preferentially grow inside the agar or agarose, as described by Han et al. (2014). Time-lapse microscopy of *C*. *glutamicum* again showed growth arrest with PenG and the emergence of small L-forms from the parental walled cells and proliferation in a typical L-form manner on addition of lysozyme (Movie S7).

### Contrasting Effects of β-Lactams on Gram-Negative Bacteria

**Figure S5:**
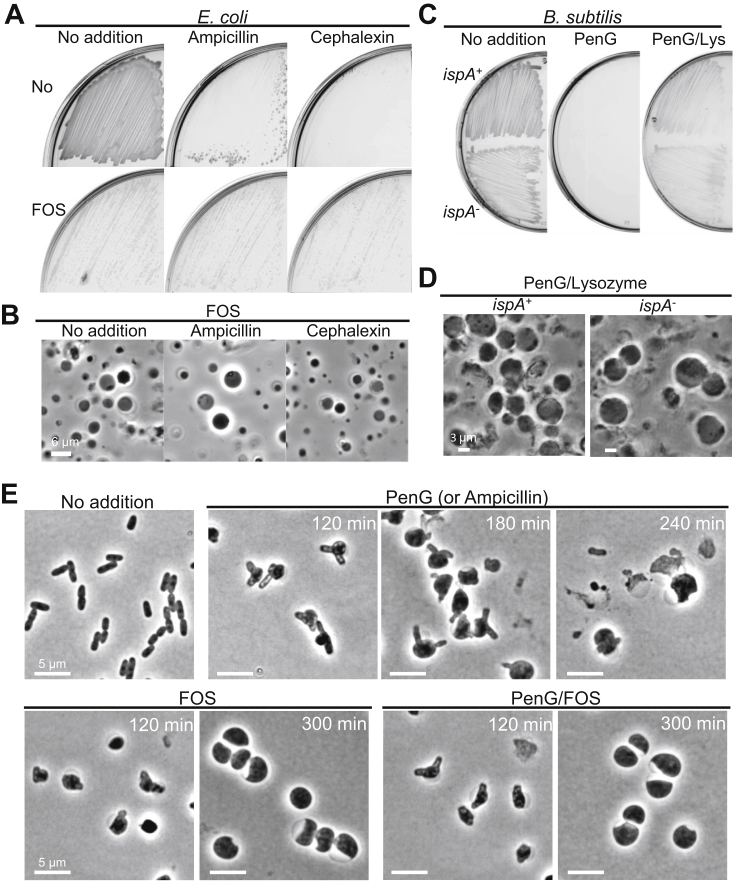
Effects of β-Lactam Antibiotics on *E. coli* L-Form Switch, Related to Figure 4 (A and B) (A), *E. coli* wild-type strain BW25113 was streaked on NA/MSM plates with (lower panels, FOS) or without 200 μg/ml fosfomycin (upper panels, No), in the presence of 100 μg/ml ampicillin (middle) or 100 μg/ml cephalexin (right), as indicated, and incubated at 30°C for 3 days under anaerobic conditions because this generates much more robust L-form growth (Kawai et al., 2015). (B), PC micrographs of cells taken from the plates shown in (A, FOS). (C and D) (C), *B. subtilis* wild-type strains (168CA) and RM81 (*ispA*^-^) were grown on NA/MSM plates with or without 200 μg/ml PenG, and 100 μg/ml lysozyme (Lys), as indicated, and incubated at 30°C for 2-3 days under anaerobic conditions. (D), PC micrographs of cells taken from the plates shown in (D, PenG/Lys). (E) *E. coli* wild-type strain BW25113 was grown in NB/MSM liquid with or without 100 μg/ml PenG and/or 100 μg/ml fosfomycin (FOS) at 30°C under aerobic conditions.

To test whether these observations held also for Gram-negative bacteria, we examined the effects of antibiotics affecting PG precursor synthesis (fosfomycin) or assembly (β-lactams) on *E. coli* under isotonic conditions (Figures S5A and S5B). In contrast to the Gram-positives, treatments with β-lactams did not inhibit L-form growth induced by fosfomycin (Figures S5A and S5B). Note that the *E. coli* experiments were carried out under anaerobic conditions because this generates much more robust L-form growth, as described previously (Kawai et al., 2015). Figures S5C and S5D show that anaerobic conditions made no difference to our findings for *B. subtilis* and indeed, as shown previously, the anaerobic conditions enabled L-form growth in *ispA*^+^ cells. Consistent with a lack of the β-lactam-induced inhibition in L-form growth, treatment with a β-lactam resulted in dramatic shape changes, predominantly bulging, and culminated with most cells lysing (Figure S5E, PenG). Treatment with fosfomycin, as we reported previously, led to a gradual and fairly regular rounding and eventually to the L-form state (Figure S5E, FOS). Crucially, when fosfomycin and β-lactam treatments were combined, the cells took on the regular rounded and stable appearance of fosfomycin-treated cells (Figure S5E, PenG/FOS) rather than following the lethal β-lactam pathway. These results are in principle consistent with the findings of Cho et al. (2014) in which β-lactams were shown to kill, at least in part, by a futile cycle of PG precursor synthesis and turnover, which can be blocked by inhibition of PG precursor synthesis.

### Promotion of L-Form Switching by the Host Environment

**Figure 5:**
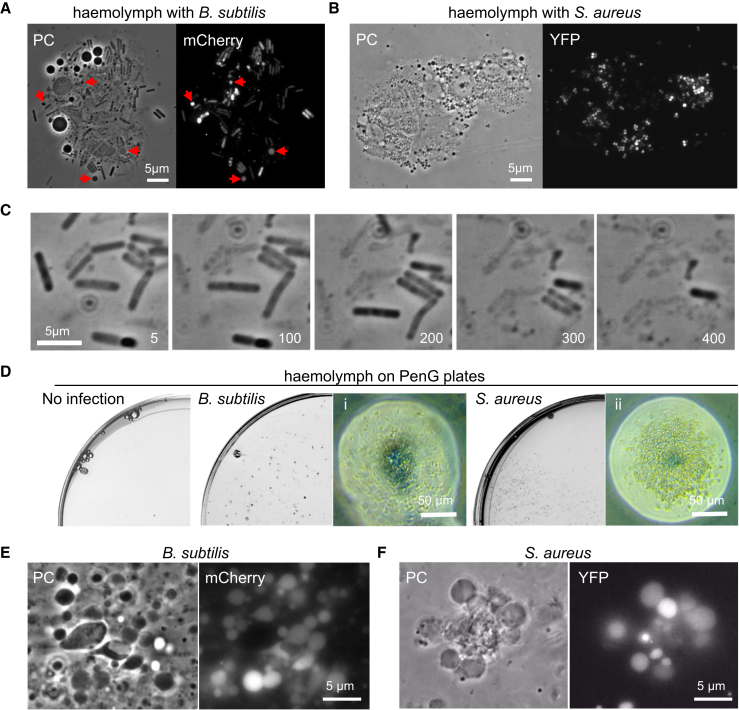
L-Form Switch Is Induced in Host Environment (A and B) L-form induction in the *G. mellonella* larvae. PC micrographs and the corresponding fluorescent images of hemocytes extracted from the *G. mellonella* larvae infected for 3 hr with walled *B. subtilis* (strain YK2267; *ispA*^−^*aprE*::*mCherry*) (A) and *S. aureus* (strain YK2310; RN4220 pYFP) (B). Examples of spherical *B. subtilis* cells are shown by the red arrows in (A). (C) Effects of PenG on walled *B. subtilis* in the larvae hemolymph on osmoprotective medium (NA/MSM). Numbers in the bottom right corner of each frame represent time (min) elapsed in an example movie from time-lapse experiments. (D and E) The larval hemolymph samples with (middle, *B*. *subtilis*; right, *S. aureus*) or without bacterial infection (left) were placed on NA/MSM plates containing 200 μg/mL PenG and incubated at 30°C for 2–3 days. Examples of L-form colony morphologies are shown in i (*B. subtilis*) and ii (*S*. *aureus*). (E) PC micrographs and the corresponding fluorescence images of *B. subtilis* (left), and *S*. *aureus* (right) L-forms were taken from the plates shown in (D).

Lysozyme, which is highly conserved throughout the animal kingdom, contributes importantly to innate immune defense against bacteria. It is present in body secretions including tears, mucus, saliva, and milk, and cells of the innate immune system such as macrophages and polymorphonuclear neutrophils produce large amounts of lysozyme (Ragland and Criss, 2017). Several old reports suggested that exposure of bacteria to macrophages may contribute to the induction of L-forms *in vivo* (Domingue and Woody, 1997). To test this, we challenged larvae of the wax moth (*Galleria mellonella*), an increasingly widely used simple animal model, which possesses an innate immune system containing phagocytic cells analogous to human macrophages (Tsai et al., 2016), with *B. subtilis* and *S. aureus* strains tagged with fluorescent fusions. After 3-hr exposure to walled bacteria, larvae were sacrificed and their hemolymph was subjected to fluorescence microscopy. Bacterial cells were observed associated with larval phagocytes (Figures 5A and 5B). Many of the *B. subtilis* cells were rod-shaped indicating that they retained an intact cell wall (Figure 5A). Addition of PenG induced a rapid cell lysis for those rods (Figure 5C), similar to the effects of PenG shown above (Figure 1G). We also observed many spherical *B*. *subtilis* cells, both associated with the hemocytes and suspended in the hemolymph (Figure 5A, arrows), which were probably generated due to the action of lytic enzymes produced by the larvae (Mak et al., 2010). To test their ability to grow in the L-form state, we plated the hemolymph on isotonic agar containing PenG. As expected, we observed abundant L-form colonies after 2 days of incubation (Figures 5D and 5E, *B. subtilis*). Close-ups of examples of the colonies revealed the typical “fried-egg” L-form colony morphology (Figure 5D, i). Similar results were obtained with *S. aureus* (Figures 5D and 5F, *S. aureus*). No colonies had appeared after 2 days for a control hemolymph incubated identically but without bacterial infection (Figure 5D, left panel). On the basis of these results, it appears that immune effectors in the host environment, presumably including lysozyme, can drive an L-form transition that could contribute to β-lactam tolerance.

### Mammalian Macrophages Induce L-Form Switching

**Figure 6:**
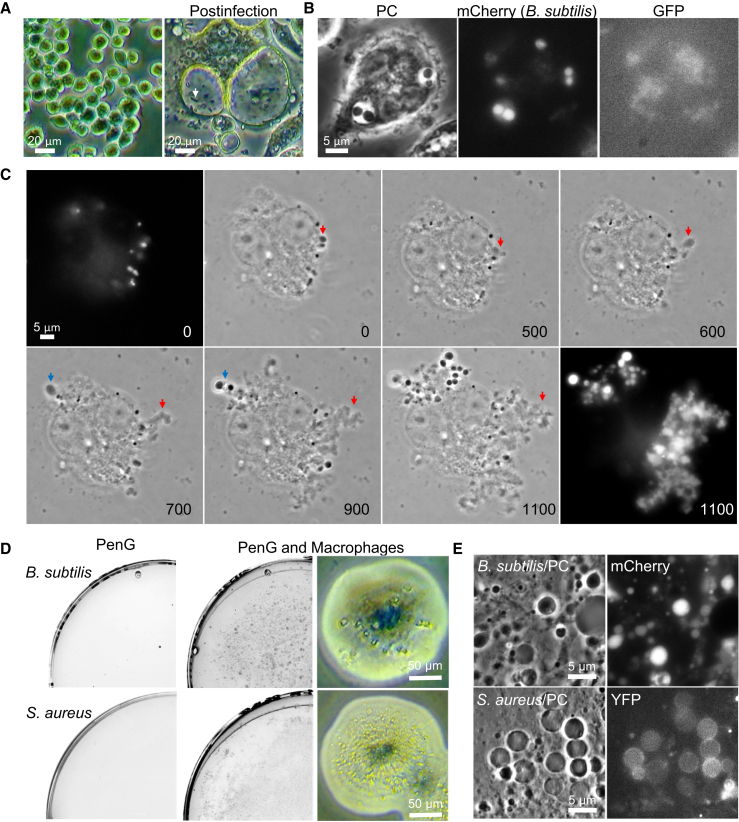
Macrophages Promote an L-Form Switch (A and B) Macrophage activation by interaction with *B*. *subtilis* walled cells. (A) The left panel shows a typical image of murine macrophage-like RAW-Blue cells in DMEM medium. After overnight interaction with *B. subtilis* walled cells (strain YK2267; *ispA*^−^*aprE*::*mCherry*), the activated macrophages were greatly enlarged and formed pseudopodia and phagocytic vacuoles (right panel). An example of a rod-shaped *B. subtilis* cell inside a vacuole is shown by the arrow. (B) PC micrograph of spherical *B. subtilis* cells inside the vacuoles (left). The middle panel shows the corresponding mCherry image, and the right panel is a GFP image to demonstrate that fluorescence is specific to the mCherry. (C–E) Effects of macrophages on L-form growth. (C) The overnight mixed culture of murine macrophages and *B. subtilis* cells in DMEM macrophage medium (no PenG) was placed on a NA/MSM micro-well dish with 200 μg/mL PenG, and incubated at 30°C for several hours before time-lapse imaging. Individual PC micrograph frames are extracted from Movie S8. Numbers in the bottom right corner of each frame represent time (min) elapsed in the movie. The top left (0) and bottom right (1,100) corner panels shows the corresponding mCherry images. Examples of L-form emergence are shown by arrows. (D) The overnight cultures of *B. subtilis* (upper) or *S*. *aureus* (lower) cells with (middle, and right for high magnification) or without macrophages (left) were placed on NA/MSM plates with 200 μg/mL PenG, and incubated at 30°C for 2–3 days. (E) PC micrographs and the corresponding fluorescent images of *B. subtilis* (upper) and *S*. *aureus* (lower) L-forms cells were taken from the plates shown in (D) (PenG and Macrophages). See also Figure S6.

To test for L-form induction in mammalian phagocytic cells, we challenged murine macrophage-like RAW-blue cells with walled cells of a *B. subtilis* strain that was tagged with mCherry. After overnight incubation, we observed a significant degree of activation of the macrophages, as judged by the formation of pseudopodia and phagocytic vacuoles (Figure 6A). Many *B. subtilis* cells had been phagocytosed: some were rod-shaped indicating that they retained an intact cell wall (Figure 6A, arrow). However, many of the *B. subtilis* cells had a spherical morphology in macrophages (Figure 6B), presumably due to the action of lytic enzyme(s) made by the macrophages (the negligible signal in the GFP channel shows that the mCherry signal was specific). Spherical *B. subtilis* cells were also seen outside the macrophages (presumably due to secreted lytic activity) (data not shown). The culture was transferred for time-lapse microscopy onto isotonic agar with PenG, which should support L-form growth but was detrimental to the macrophages. Figure 6C and Movie S8 show a typical example of the time-lapse imaging. Remarkably, *B. subtilis* cells were able to escape from the dead macrophage and propagate in a typical L-form manner, as described above. We plated the mixed culture of murine macrophages and *B. subtilis* cells on NA/MSM plates containing PenG and observed a lawn of small colonies, which emerged within 2 days (Figures 6D and 6E, *B. subtilis*). These colonies had a typical L-form colony morphology in close up (Figure 6D, right). No colonies had appeared after 2 days for a control bacterial culture incubated identically but without macrophages (Figure 6D, left). We repeated the macrophage experiments with *S*. *aureus* tagged with YFP, and the results were similar to those for *B. subtilis* (Figures 6D and 6E, *S. aureus*). These results strongly support the idea that immune effectors, such as lysozyme, produced by macrophages, can induce L-form switching of both *B. subtilis* and *S. aureus*, which then bypasses the antibacterial activity of PenG.

### Macrophages Can Protect Bacteria from β-Lactam Killing

**Figure 7:**
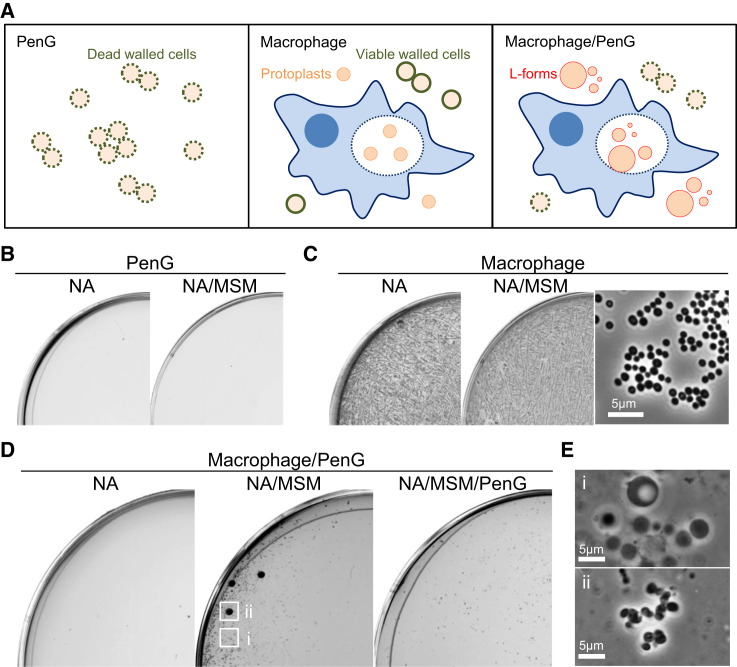
Bacterial Cells Evade Antibiotic Action of PenG by Interacting with Macrophages (A) Schematic representation for effects of macrophages on *S. aureus* survival in the presence of PenG. (B–E) Effects of macrophages on *S. aureus* survival and L-form growth in the presence of PenG under tissue culture conditions. (B) The overnight culture of *S. aureus* walled cells in DMEM containing 200 μg/mL PenG was plated on NA and NA/MSM plates and incubated at 30°C for 7 days. (C) The overnight culture of macrophages and *S. aureus* walled cells in DMEM (no PenG) was plated on NA and NA/MSM plates, and incubated at 30°C. PC micrograph of *S*. *aureus* walled cells (right) was taken from the NA/MSM plate (middle). (D) The overnight culture of macrophages and *S. aureus* walled cells in DMEM with PenG (preceded by 5 hr incubation without PenG) was plated on NA, NA/MSM, and NA/MSM with PenG plates and incubated at 30°C. (E) PC micrographs of *S*. *aureus* L-forms (i) and walled cells (ii) were taken from the NA/MSM plate shown in (D) (middle). We confirmed walled or L-form state by streaking on hypotonic plates on which only walled cells grow.

Given the above results, we wondered whether macrophages could actually protect bacterial cells from PenG action in tissue culture conditions. We challenged macrophages with *S. aureus* walled cells and incubated for 5 hr, followed by the addition of PenG to the culture. After 24 hr incubation in the presence of PenG, the culture was plated on various media and colony formation was monitored for 7 days. In a control culture incubated without macrophages (Figure 7A, left), no colonies had appeared on either isotonic or hypotonic NA plates (Figure 7B), indicating efficient cell killing by PenG and no generation of L-forms. In the culture with macrophages but without PenG (Figure 7A, middle), abundant walled cell colonies were observed on both hypotonic and isotonic plates (Figure 7C). In contrast, for the culture with both macrophages and PenG (Figure 7A, right), many small L-form colonies started to appear on isotonic plates after 2–3 days (Figures 7D, middle, and 7E, i) but not on hypotonic plates (Figure 7D, left). The longer incubation also promoted the emergence of a few larger colonies on isotonic plates (Figure 7D, ii), which appeared to contain walled cells (Figure 7E, ii) and indeed were able to grow on hypotonic medium. Because L-forms are known to be able to regenerate cell wall in the absence of selection pressure such as PenG (Kawai et al., 2014), they probably represent L-forms that had reverted to the walled state. Indeed, we did not detect large colonies on isotonic plates containing PenG (Figure 7D, right). These results showed that interaction with macrophages can protect bacterial cells from β-lactam killing, apparently by promoting an L-form switch.

### The L-Form Switch Is Driven by Host Lysozyme and Possibly Other Lytic Factors

**Figure S6:**
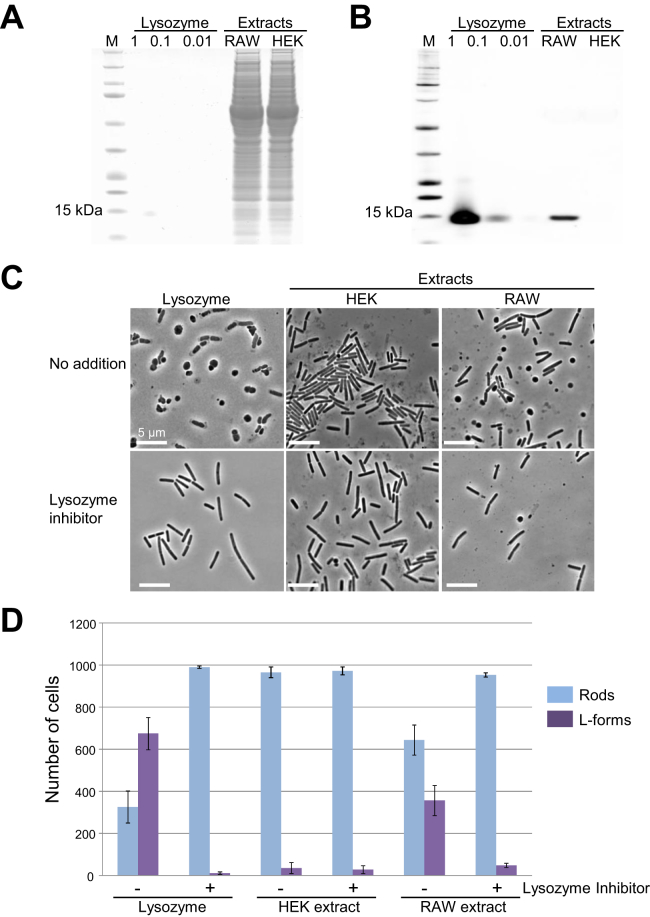
L-Form Switch by Host Lysozyme in Macrophage Extract, Related to Figure 6 (A and B) Visualization of lysozyme in whole cell extracts of macrophage-like RAW-Blue cells by SDS-PAGE and western blot analysis. (A), Coomassie staining of purified human lysozyme (1, 0.1 and 0.01 μg), and whole cell extracts of RAW-Blue cells (RAW) and human embryonic kidney cells (HEK), as indicated. M represents molecular weight marker. (B), western blot analysis with monoclonal anti-lysozyme antibody. (C and D) Effects of lysozyme inhibitor on L-form switch in macrophage extract. (C), Exponentially growing cells of *B. subtilis* strain RM81 (*ispA*^-^) were incubated with purified human lysozyme (Lysozyme), HEK extract or RAW extract (RAW) in the presence or absence of lysozyme inhibitor (N,N’,N”-triacetylchitotriose), as indicated. (D), The relative frequency of L-form switching induced by purified lysozyme (Lysozyme), HEK extract and RAW extract in the presence or absence of lysozyme inhibitor. The L-form switch was analyzed using similar images to (C). Cells that undergone the switch are marked with purple (L-forms) and cells that did not undergo the switch are marked in blue (Rods), as indicated. 1000 cells were counted for each experiment. The columns represent the mean of 3 experiments with purified lysozyme and HEK lysate and average of 5 experiments for RAW lysate. Error bars represent standard deviation.

To confirm that macrophage-derived lysozyme was contributing to the apparent L-form switch, we made extracts from cultures of macrophages and from a lysozyme-non-producing cell type, human embryonic kidney (HEK) cells, and tested them for production of lysozyme by western blotting and ability to cause rounding of *B. subtilis* walled cells. As shown in Figures S6A and S6B, lysozyme was readily detected in macrophage extract but not in HEK cell extract. Macrophage extract (but not significantly HEK cell extract) also caused rounding of *B. subtilis* cells and crucially, this was greatly reduced by the presence of a specific inhibitor of lysozyme, N,N′,N″-triacetylchitotriose (NNNT) (Ragland and Criss, 2017) (Figures S6C and S6D). As a control, we showed that purified lysozyme also generated rounding of *B. subtilis* cells, and in this case, inhibition by NNNT was complete. It seems possible that the residual rounding activity by macrophage and HEK extracts in the presence of NNNT is due to one or more alternative lytic factors, such as PGLYRP2 (Dziarski and Gupta, 2017).

## Discussion

### Contrasting Effects of PG Precursor and Assembly Inhibitors

We previously showed that the inhibition of PG precursor synthesis (e.g., by fosfomycin [FOS] or D-cycloserine [DCS]) promotes a walled to L-form transition in a wide range of bacteria, including various Gram-positives and a model Gram-negative (*E. coli*), and representing both free living and pathogenic bacteria (Mercier et al., 2013, Mercier et al., 2014). It is striking (Figures 1E, 3D, and S5E) that treatment with FOS or DCS, which should block precursor synthesis rapidly and completely, is followed by continued expansion of the cells, leading to bulging and then L-form escape. Unless inhibition is incomplete, which seems unlikely given the high doses of inhibitor, or the precursor pools are unexpectedly large, this expansion must be due to unchecked autolytic activity in the existing cell wall. In contrast to this effect, treatment with β-lactams generally results in an immediate arrest in growth and only limited shape changes, with no L-form release, for all of the Gram-positives tested (Figures 1F, 1G, 4D, and S4) (but not *E. coli*; see below). Therefore, inhibition of PG synthesis at the assembly level presumably also blocks autolytic activity, and hence L-form release. The rescue of L-form growth by lysozyme provides strong support for this view.

β-lactams have many target TPases in the cell. Among these, the class A PBPs also have GTase activity, which is needed, together with TPase activity, to synthesize PG. Recent exciting reports have revealed that the widely conserved RodA protein (and probably the wider family of SEDS proteins to which it belongs) also has GTase activity, and this protein is essential for the elongation of rod-shaped cells, working in conjunction with the MreB cytoskeleton and specific class B (monofunctional TPase) PBPs (Cho et al., 2016, Emami et al., 2017, Meeske et al., 2016). It is not yet clear why rod-shaped bacteria have two distinct major PG synthetic systems, but our results show clearly that β-lactam inhibition of autolytic activity occurs through the class A PBP pathway. Thus, MOE, a specific inhibitor of the GTase of class A PBPs elicits effects similar to those of β-lactams, as does deletion of the 4 class A PBP genes, whereas deletion of *rodA* does not block, but rather promotes cell bulging (Figure 3E). Although it is not yet clear how class A PBP and autolytic activities are coupled, our results provide interesting new insights into the differential roles of the two PG synthetic systems.

### Different Responses of Gram-Positive and Gram-Negative Bacteria

The distinction between the responses of Gram-negative and Gram-positive bacteria to β-lactam treatment were first pointed out long ago (Rodríguez-Tébar et al., 1982). However, the molecular basis for these differences are still not well understood. Both classes of organism respond similarly to inhibition of precursor synthesis, in undergoing bulging and under appropriate conditions, L-form growth (Mercier et al., 2014 and above). Bulging and shape changes were also evident for *E. coli* treated with β-lactams, presumably due to ongoing autolytic activity, but despite these morphological changes, L-form growth did not occur and the cells died (Figure S5). Although other explanations are possible, such as oxidative stress, it is not clear why this would not also occur for precursor synthesis inhibitors. Recent work from Cho et al. (2014) has shown that a futile cycle of lipid II synthesis and turnover can contribute to β-lactam-induced cell death in *E. coli*. This would be consistent with our observation that inhibition of precursor synthesis by FOS protected cells from β-lactam-induced cell death (Figure S5). This result was again strikingly different from, indeed the reverse of, the response of the Gram-positive bacteria, in which the β-lactam “trumped” the precursor inhibitor (DCS) (e.g., Figure 1). However, it is pertinent to note that other Gram-negative pathogens, including *Vibrio cholera*, have been reported to respond to β-lactam antibiotics by switching to a rounded stable form (although not a proliferating L-form state), requiring continued cell wall hydrolase activity (Dörr et al., 2015). Clearly, further work will be needed to understand the general principles of how β-lactams impact on L-form switching and maintenance in Gram-negative bacteria.

### Host Lytic Enzymes Can Promote the L-Form Switch

As mentioned above, the notion that the β-lactam effect in Gram-positive bacteria is due to a block in autolytic activity is strongly endorsed by the fact that exogenous lysozyme can rescue L-form generation. There are many potential sources of lytic enzymes in nature. We have shown that macrophages and the hemolymph of *G. mellonella*, at least, contain molecules that can bypass the PenG-block to the L-form switch. We showed directly that the macrophage samples contained lysozyme (Figure S6), consistent with expectation (Ragland and Criss, 2017) and this probably contributes much of the lytic activity needed for the L-form switch. However, the residual activity seen in the presence of a specific inhibitor of lysozyme points to the presence of other lytic factors. PGLYRP2 protein has previously been identified as an innate immune effector with PG amidase activity, but immune cells are thought to make a plethora of different effector molecules, either constitutively or in response to infection (Dziarski and Gupta, 2017). Thus, more work on characterizing their specific enzymatic activities on bacterial components would seem warranted. Whatever the specific mechanisms involved, it seems remarkable that macrophages can be protective in β-lactam challenge experiments.

### Cell Wall-Deficient Bacteria in Macrophages Can Retain Viability

Given the battery of innate immune weapons that immune cells can elaborate, including ROS, antimicrobial peptides, acidification, and hydrolytic enzymes, it is generally assumed that, with the exception of “professional” intracellular pathogens such as *Listeria* or *Shigella*, most bacteria phagocytosed by macrophages will be killed. However, our results show definitively that bacterial cells that have been damaged and are morphologically aberrant can nevertheless retain viability and grow if conditions are suitable. We have seen this survival both in a macrophage cell line and in the hemolymph of *G. mellonella*, including bacteria engulfed by phagocytic cells (Figures 5 and 6). It therefore seems likely that the switch into a viable L-form-like state will occur at a significant frequency whenever Gram-positive bacteria encounter phagocytic cells or environments containing lytic enzymes, and crucially, this morphological transition does not necessarily signify bacterial cell death. It remains to be determined how long cell wall-deficient bacteria can survive inside the phagocytic cells (or other *in vivo* niches) and what are the characteristics of the individual phagocytes that would allow long-term persistence of L-forms.

### Implications for Persistent or Recurrent Infection

L-forms are completely resistant to β-lactam antibiotics, and thus, the L-form switch could help bacteria to survive exposure to antibiotics in infected humans. There is a long history of reports (albeit frequently anecdotal) of the recovery of L-forms from antibiotic-treated patients (Clasener, 1972, Domingue, 2010, Domingue and Woody, 1997, Errington et al., 2016, Onwuamaegbu et al., 2005). In principle, many tissue locations are sufficiently osmoprotective to enable the survival of L-forms *in vivo*. Crucially, these conditions are not equivalent to the hypotonic agar plating methods frequently used to investigate antibiotic action. Our macrophage experiments showed directly that the action of these cells can protect Gram-positive bacteria from killing by high concentrations of PenG under semi-physiological conditions (Figure 7), and it seems highly likely that this occurs by the conversion of walled bacteria into an L-form state. If the L-forms avoid innate immune killing, they could survive prolonged exposure to cell wall-active antibiotics. Several mechanisms enabling the recurrence of infection have been described. The most common involve entry of cells, called persisters, into a dormant, non-growing state (Fisher et al., 2017, Harms et al., 2016). Many antibiotics fail to kill such non-growing cells. However, unlike these dormant persisters, L-forms remain viable and can continue to proliferate in the presence of antibiotic. At the end of antibiotic treatment, L-forms can revert to the walled state (Domingue and Woody, 1997, Kawai et al., 2014, Mercier et al., 2014) (Figures 7D and 7E), enabling the cells, in principle, to reinitiate a full blown infection. Being highly pleomorphic and flexible, L-forms may also gain advantage and persist by invading tissues and physiological niches that are inaccessible to walled cells. Given the effectiveness of β-lactam antibiotics in most clinical situations, it is likely that L-forms become problematical only under certain circumstances (e.g., in elderly and or immunocompromised individuals) where treatment failures are not uncommon. Although much more work is needed to clarify the clinical importance of L-forms, our results provide important new insights into the effects of innate immune molecules such as lysozyme on antibiotic evasion by bacteria.

Finally, it is ironic that Fleming’s two great antibacterial discoveries, lysozyme (Fleming, 1922) and penicillin (Fleming, 1929), turn out to act antagonistically under certain circumstances.

## STAR★Methods

### Key Resources Table

**Table:** 

REAGENT or RESOURCE	SOURCE	IDENTIFIER
**Antibodies**		

Rabbit monoclonal Anti-Lysozyme antibody [EPR2994(2)]	Abcam	Cat#ab108508; RRID:AB_10861277
Goat Anti-Rabbit IgG HRP	Sigma	Cat#0545

**Bacterial and Virus Strains**		

*Bacillus subtilis* 168CA (wild-type) *trpC2*	Lab. stock	N/A
*Bacillus subtilis* CU267 (ϕ105MU3) *trpC2 ilvB2 leuB16* (ϕ105MU3) Ω(*lacZ cat*)3	Thornewell et al., 1993	N/A
*Bacillus subtilis* RM81 *trpC2 xseB::Tn kan* (*ispA*^-^)	Mercier et al., 2013	N/A
*Bacillus subtilis* LR2 *trpC2 P*_*xyl*_-*murE cat ispA*^-^	Mercier et al., 2013	N/A
*Bacillus subtilis* YK1354 *trpC2 xseB::Tn kan* (*ispA*^-^) *rodA::kan*	Kawai et al., 2011	N/A
*Bacillus subtilis* YK2267 *trpC2 xseB::Tn kan* (*ispA*^-^) *aprE*::*mCherry spc*	Kawai et al., 2015	N/A
*Bacillus subtilis* YK2288 *trpC2 P*_*xyl*_-*murE cat xseB::Tn kan* (*ispA*^-^) *ΩcwlO::*pMutin4-*erm*-*P*_*spac*_-*cwlO*	This work	N/A
*Bacillus subtilis* YK2289 *trpC2 P*_*xyl*_-*murE cat xseB::Tn kan* (*ispA*^-^) *ΩcwlO::*pMutin4-*erm*-*P*_*spac*_-*cwlO lytE::cat*	This work; Domínguez-Cuevas et al., 2012	N/A
*Bacillus subtilis* YK2292 *trpC2 P*_*xyl*_-*murE cat ispA*^-^*P*_*spac*_-*rodA kan*	Emami et al., 2017	N/A
*Bacillus subtilis* YK2294 *trpC2 xseB::Tn kan* (*ispA*^-^) *amyE::hbsU-gfp cat*	Leaver et al., 2009	N/A
*Bacillus subtilis* YK2313 *trpC2 xseB::Tn kan* (*ispA*^-^) Δ6 (prophages^-^)	Westers et al., 2003	N/A
*Bacillus subtilis* YK2314 *trpC2 xseB::Tn kan* (*ispA*^-^) Δ6 (prophages^-^) *P*_*xyl*_-*murE spc*	This work	N/A
*Bacillus subtilis* YK2344 *trpC2 xseB::Tn kan* (*ispA*^-^) Δ4 (Δ*ponA*, *pbpD*, *pbpF* and *pbpG*)	Emami et al., 2017	N/A
*Staphylococcus aureus* RN4220	Kreiswirth et al., 1983	N/A
*Staphylococcus aureus* YK2310 RN4220 pGL485-yfp	Steele et al., 2011	N/A
*Corynebacterium glutamicum*	Lab. stock	ATCC13032
*Enterococcus faecium*	Lab. stock	ATCC19434

**Chemicals, Peptides, and Recombinant Proteins**		

Lysozyme form human neutrophiles	Sigma-Aldrich	Cat#L8402
Lysozyme from from chicken egg white	Sigma-Aldrich	Cat#L6876
Lysostaphin from *Staphylococcus staphylolyticus*	Sigma-Aldrich	Cat#L7386
N,N′,N″-Triacetylchitotriose	Sigma-Aldrich	Cat#T2144
Quanti-Blue reagent	InvivoGen	Cat#rep-qb2

**Experimental Models: Cell Lines**		

RAW-Blue cells	InvivoGen	Cat#Raw-sp; RRID:CVCL_X594
Flp-In 293 T-REx cells	Thermo Fisher	Cat#R78007; RRID:CVCL_U427

**Experimental Models: Organisms/Strains**		

Truelarv *Galleira mellonella*	BioSystems Technology	N/A

**Oligonucleotides**		

cwlO-F GAAGAATTCCATTCCTCGTAGAGTATG	This paper	N/A
cwlO-R GGAGGATCCCTTGAGATCCGCCGCTTTC	This paper	N/A

**Recombinant DNA**		

pM4-*P*_*spac*_-*cwlO* plasmid	This paper	N/A

**Other**		

35 mm sterile glass bottom microwell dishes	Ibidi GmbH	Cat#80136

### Contact for Reagent and Resource Sharing

Further information and requests for resources and reagents should be directed to and will be fulfilled by the Lead Contact, Jeff Errington (jeff.errington@ncl.ac.uk).

### Experimental Model and Subject Details

#### Bacterial Strains and Growth Conditions

The bacterial strains in this study are listed in the Key Resources Table. Nutrient agar (NA, Oxoid) or nutrient broth (NB, Oxoid) were used for bacterial growth at 30°C. Müeller Hinton agar (Sigma-Aldrich) was used for growth of *E*. *faecium*. Supplements, 1 mM IPTG or 1% xylose were added when required. Bacterial L-forms were grown in osmoprotective medium composed of 2x magnesium-sucrose-maleic acid (MSM) pH7 (40 mM MgCl_2_, 1 M sucrose, and 40 mM maleic acid) mixed 1:1 with 2x NB or 2x NA at 30°C. When necessary, antibiotics were added to media at the following concentrations: 1 μg/ml erythromycin, 200 μg/ml D-cycloserine, 200 μg/ml fosfomycin, 200 μg/ml PenG, 100 μg/ml ampicillin, 50 μg/ml MOE, 100 μg/ml cephalexin and/or 1 μg/ml 8J (FtsZ inhibitor) (Adams et al., 2011). Anaerobic growth condition was maintained using anaerobic atmosphere generation bags (AnaeroGenTM, Oxoid) in an anaerobic jar for growth on plates. 10 mM Nitrate and 0.1% glutamic acid were added for *B. subtilis* growth under anaerobic conditions. For reasons that are not yet clear, the L-form switch induced by PenG and lysozyme worked well on solid or semi solid agar, but not or less efficient in liquid conditions.

#### Cell lines

RAW-Blue cells (InvivoGen Cat#raw-sp, RRID:CVCL_X594) are derived from the murine adult male RAW 264.7 macrophages with chromosomal integration of a secreted embryonic alkaline phosphatase (SEAP) reporter construct inducible by NF-κB and AP-1. The cell line was authenticated by RT-PCR. Flp-In 293 T-REx cells (Thermo Fisher Cat#R78007, RRID:CVCL_U427) are derived from human female embryonic kidney cell line HEK293 and are designed for rapid generation of stable cell lines that ensure homogeneous expression of protein of interest from a Flp-In expression vector. These cells contain a single stably integrated FRT site at a transcriptionally active genomic locus. Both cell lines were maintained in Dulbecco’s Modified Eagle’s medium (DMEM, Sigma Cat#D6429) supplemented with 5% fetal bovine serum (FBS) at 37°C and 5% CO_2._

#### *Galleria mellonella* insect model

*Galleria mellonella* TrueLarv (Biosystems technology) are healthy, weight and aged defined larvae in the sixth and final instar stage of development. While at this stage, it is not possible to sex the larvae using external markers. Weighing between 0.18 and 0.35 g each, the larvae are surface-sterilized and delivered in sterilized pots, minimizing the likelihood of external contaminants. The larvae are from an inbred colony that does not receive either antimicrobials or growth hormones in its feed. *G. mellonella* can be kept in their pots at 15°C for up to two weeks after delivery without any requirement for food or water. There is an ongoing genome sequencing program for the breeding colony, therefore genotype is not specified. The larvae were ordered as needed from the supplier and used within a week from delivery. Animals were not used for other procedures.

### Method Details

#### Construction of IPTG-Inducible *cwlO*

The first 200∼300 bp of the *cwlO* gene containing Shine-Dalgarno sequence was amplified by PCR from genomic DNA of the wild-type strain 168CA using the primers cwlO-F and cwlO-R (Key Resources Table), then cloned between the *Eco*RI and *Bam*HI sites of plasmid pMutin4 (Vagner et al., 1998), creating pM4-*P*_*spac*_-*cwlO*. The resulting plasmids were introduced into *B*. *subtilis* to generate YK2288 and YK2289 (Key Resources Table). In those strains, the full-length *cwlO* gene is expressed from the IPTG-inducible promoter *P*_*spac*_. DNA manipulations and transformations were carried out using standard methods.

#### Microscopic Imaging

For snapshot live-cell imaging, cells were mounted on microscope slides covered with a thin film of 1% agarose in water, or NB/MSM for L-forms. Images were acquired with a Sony Cool-Snap HQ2 cooled CCD camera (Roper Scientific) attached to a Zeiss Axiovert 200M, or a Rolera EM-C2 (Q-imaging) camera attached to a Nikon TiE microscope, and analyzed using Metamorph (Molecular Devices). For time lapse imaging, 300 μL of exponentially growing bacterial cells in NB/MSM or the mixed culture of RAW-Blue macrophages and bacterial cells in Dulbecco’s Modified Eagle’s medium (DMEM) were placed on 35 mm sterile glass bottom microwell dishes (ibidi GmbH, Munich, Germany), which were pre-coated with 2 mg/ml bovine serum albumin (BSA), and incubated for 5 min. For the depletion of MurE and/or CwlO, using *P*_*xyl*_-*murE* and *P*_*spac*_-*cwlO*, exponential cell cultures in NB/MSM with xylose and/or IPTG at 30°C were diluted 10-fold in fresh NB/MSM (without xylose and/or IPTG). After incubation for ∼2 hr at 30°C, the cells were placed on the microwell dishes. The dishes were centrifuged at 100 g for 2 min using a Beckman Allegra X-12R centrifuge. Non-adherent cells were removed, and a thin layer of NB/MSM with 0.2% agar bacteriological (Oxoid) was placed on the top of the dishes. The dishes were placed on the microscope stage at 30°C. Images were acquired with a Sony Cool-Snap HQ2 cooled CCD camera (Roper Scientific) attached to a Zeiss Axiovert 200M, and analyzed using Metamorph (Molecular Devices). Low magnification images of bacteria interacting with macrophages as well as L-form colonies were obtained using Moitc AE200 inverted microscope equipped with 20x phase contrast objective and MOTICAM digital camera. Pictures and videos were prepared for publication using ImageJ (https://imagej.nih.gov/ij) and Adobe Photoshop.

#### Protoplast preparation

Protoplasts were prepared by adding 100 μg/ml lysozyme (for *B. subtilis*) or 5 μg/ml lysostaphin (for *S. aureus*) to exponentially growing bacterial cultures in NB/MSM (OD_600nm_ of 0.1-0.2) at 37°C, and incubated for 30-60 min.

#### Bacterial Infection Conditions

RAW-Blue cells were seeded the day before for bacterial infection experiments at 0.5 × 10^6^ cells per ml DMEM 5% FBS medium in 25 cm angled neck flasks (NUNC). The macrophages were challenged at a multiplicity of infection (MOI) 100 with *B. subtilis* (strain YK2267; *ispA*^-^ *aprE*::*mCherry*) and *S. aureus* (strain YK2310; RN4220 pYFP) *at* MOI 1 (or MOI 5 for experiments in the presence of PenG) in 5 mL of DMEM medium and incubated overnight at 37°C and 5% CO_2_ together with control flasks containing bacteria only. Where indicated, PenG at a final concentration of 200 μg/ml was added following 5 h co-incubation without the antibiotic. Adherent cells were dislodged with a scrapper and 100 μl cells were plated onto NA/MSM containing 200 μg /ml PenG plates, or used for microscopy experiments.

*Galleria mellonella* larvae were injected in the left posterior proleg with 10 μL inocula of *B. subtilis* (strain YK2267; *ispA*^-^ *aprE*::*mCherry*) or *S. aureus* (strain YK2310; RN4220 pYFP) containing 8 × 10^9^ CFU/ml using microliter syringe (Hamilton) and incubated for 3 h at 37°C. Following the incubation, larvae that did not show signs on melanisation were placed in 1.5 mL microfuge tubes on ice for 30 min. After this time two posterior segments were removed with surgical scissors and the haemolymph was extracted into the microfuge tubes. 1 μL of the hemolymph was subjected to microscopy and 20 μL were placed on NA/MSM plates containing 200 μg/ml PenG and incubated for 2 days at 30°C.

#### RAW and HEK lysate preparation

RAW-Blue (RAW) and Flp-In 293 T-Rex (HEK) cells were seeded the day before lysate preparation at 0.5 × 10^6^ cells per ml DMEM 5% FBS medium in 25 cm angled neck flasks (NUNC). Following overnight incubation the cells were adjusted to 6 × 10^6^ cells in 300 μL DMEM 5% FBS media containing cOmplete Protease Inhibitor Cocktail (Roche Cat#04693159001) and sonicated on ice using Sonics Vibra-Cell sonicator (VWR) with 3 bursts of 30 s at 40% amplitude. Following sonication, the samples were cleared by centrifugation at 12,000 g for 1 min.

#### SDS-PAGE

Serial 10-fold dilutions of purified human neutrophil lysozyme (Sigma-Aldrich Cat#L8402) were prepared in sterile water at concentrations of 200 μg/ml, 20 μg/ml and 2 μg/ml. Serial dilutions of the lysozyme as well as RAW and HEK lysates adjusted to 5 mg/ml were fixed with 2x Laemmli Sample Buffer (Bio-Rad Cat#161-0737) containing 150 mM DTT, heated for 5 min at 98°C and 10 μL of each sample were loaded along 5 μL of Precision Plus Protein WesternC molecular weight standard (Biorad Cat#161-0737) onto NuPAGE 4%–12% Bis-Tris midi gel (Invitrogen Cat#WG1402BX10). The samples were resolved by running in 1x MES buffer (Life technologies Cat#NP0002) at 200 V for 45 min using XCell4 SureLock Midi-Cell Electrophoresis System (Invitrogen Cat#WR0100). The resolved gel was stained by Commassie Brilliant Blue or subjected to western blotting.

#### Western blotting

Samples resolved by SDS-PAGE were transferred using Turbo Blot system (Bio-Rad Cat#170-4155) onto PVDF membrane (Bio-Rad Cat#170-4157) which was then blocked for 1 h in 1x PBS, 2% powder milk (Marvel) and 0.005% Tween 20, followed by overnight incubation in 1x PBS, 2% powder milk (Marvel) and 0.005% Tween 20 containing 1/5000 dilution of Rabbit monoclonal Anti-Lysozyme antibody (Abcam Cat#ab108508, RRID:AB_10861277). The membrane was washed three times in 1x PBS, 0.005% Tween 20 solution and incubated for 1 h in 1x PBS, 2% powder milk (Marvel) and 0.005% Tween 20 containing 1/5000 dilution of Goat Anti-Rabbit IgG HRP antibody (Sigma Cat#0545) and 1/10,000 dilution of Precision Protein StrepTactin-HRP conjugate (Bio-Rad Cat#1610380). The samples were visualized using Pierce ECL Plus western blotting substrate (Thermo Fisher Cat#32132) and ImageQuant LAS4000 mini biomolecular imager (GE Healthcare Sciences).

#### Lysozyme inhibition assay

*B. subtilis* strain YK2267 was grown in 5 mL NA at 37°C with shaking for 2h. RAW lysate, HEK lysate or lysozyme at 0.1 mg/ml in DMEM 5% FBS were mixed 1:1 with NA/MSM in duplicates and incubated for 15 min with or without the lysozyme inhibitor N,N′,N″-Triacetylchitotriose (Sigma Cat#T2144) at a final concentration of 5 mg/ml. Approximately 3 × 10^8^ bacterial cells were placed in 1.5 mL microfuge tubes in 6 replicas and centrifuged at 12,000 g for 1 min. Supernatant was removed and the cells were resuspended in 100 μL of the lysates or the lysozyme preincubated with or without the inhibitor, followed by 30 min incubation at 37°C. The cells were subjected to phase contrast microscopy on 1% agarose NB/MSM pad.

### Quantification and Statistical Analysis

For larvae infection experiments 10 animals were used for each of three repeats (n = 30). For the lysozyme inhibition experiment the columns represent mean number of round or rod-shaped bacteria of 1000 quantified in 3 experiments (n = 3000) with purified lysozyme and HEK lysate, and mean number of round or rod-shaped bacteria out of 1000 quantified in 5 experiments (n = 5000) for RAW lysate. Error bars represent standard deviation. Mean was used to estimate the magnitude of an effect and standard deviation to estimate the distribution of the data. No other methods were used to determine whether the data met assumptions of the statistical approach.

Definition of precision measures used:

Mean is the number obtained by adding several quantities together and dividing the sum by the number of quantities.
Standard Deviation is the root mean square deviation about the mean.

## Supplemental Information

Movie S1. L-Form Switch in *B. subtilis*, Related to Figure 1E Cells (strain LR2; *P*_*xyl*_-*murE ispA*^-^) were imaged in NB/MSM with 0.2% agar (no xylose). PC images were acquired every 5 min (Numbers in the top right corner). The movie displayed at 7 frames per second. Scale bar represents 5 μm.

Movie S2. Inhibition of L-Form Switch by PenG, Related to Figure 1F Cells (strain LR2; *P*_*xyl*_-*murE ispA*^-^) were imaged in NB/MSM with 0.2% agar in the presence of PenG (no xylose). PC images were acquired every 5 min (Numbers in the top right corner). The movie displayed at 7 frames per second. Scale bar represents 5 μm.

Movie S3. Effects of Lysozyme on *B. subtilis* L-Form Growth in the Absence of LytE and CwlO, Related to Figure 2E Cells (strain YK2289; *P*_*xyl*_-*murE ispA*^-^*P*_*spac*_-*cwlO lytE*^-^) were imaged in NB/MSM with 0.2% agar (no xylose and IPTG) in the presence of lysozyme. PC images were acquired every 5 min (Numbers in the top right corner). The movie displayed at 7 frames per second. Scale bar represents 5 μm.

Movie S4. Effects of Lysozyme on *B. subtilis* L-Form Growth in the Presence of PenG, Related to Figure 2F Cells (strain RM81; *ispA*^-^) were imaged in NB/MSM with 0.2% agar in the presence of PenG and lysozyme. PC images were acquired every 5 min (Numbers in the top right corner). The movie displayed at 7 frames per second. Scale bar represents 5 μm.

Movie S5. Effects of PenG on Growth of *S. aureus*, Related to Figures 4C and 4D Cells (strain RN4220) were imaged in NB/MSM with 0.2% agar in the presence (right) or absence of PenG (left). PC images were acquired every 5 min (Numbers in the top right corner). The movie displayed at 7 frames per second. Scale bar represents 5 μm.

Movie S6. Effects of Lysostaphin on *S. aureus* L-Form Growth in the Presence of PenG, Related to Figure 4E Cells (strain RN4220) were imaged in NB/MSM with 0.2% agar in the presence of PenG and 2 μg/ml lysostaphin. PC images were acquired every 5 min (Numbers in the top right corner). The movie displayed at 7 frames per second. Scale bar represents 5 μm.

Movie S7. Effects of PenG and Lysozyme on Growth of *C. glutamicum*, Related to Figures S4 and S4D Cells (strain ATCC13032) were imaged in NB/MSM with 0.2% agar with (right) or without lysozyme (left) in the presence of PenG. PC images were acquired every 5 min (Numbers in the top right corner). The movie displayed at 7 frames per second. Scale bar represents 5 μm.

Movie S8. Effects of Macrophages on *B. subtilis* L-Form Growth in the Presence of PenG, Related to Figure 6C Mixed cells of murine macrophages and *B. subtilis* (strain YK2267; *ispA*^-^) were imaged in NB/MSM with 0.2% agar in the presence of PenG. PC images were acquired every 5 min (Numbers in the top right corner). The movie displayed at 7 frames per second. Scale bar represents 5 μm.
